# Perceived barriers related to testing, management and treatment of HCV infection among physicians prescribing opioid agonist therapy: The C‐SCOPE Study

**DOI:** 10.1111/jvh.13119

**Published:** 2019-06-11

**Authors:** Alain H. Litwin, Martine Drolet, Chizoba Nwankwo, Martha Torrens, Andrej Kastelic, Stephan Walcher, Lorenzo Somaini, Emily Mulvihill, Jochen Ertl, Jason Grebely

**Affiliations:** ^1^ Department of Medicine University of South Carolina School of Medicine ‐ Greenville and Prisma Health Greenville South Carolina; ^2^ Clemson University School of Health Research Clemson South Carolina; ^3^ Merck Canada Inc. Montreal Canada; ^4^ Merck & Co., Inc. Kenilworth New Jersey; ^5^ Department of Psychiatry Institut de Neuropsiquiatria i Addiccions Hospital del Mar Barcelona IMIM (Institut Hospital del Mar d'Investigacions Mediques) Universitat Autònoma de Barcelona Barcelona Spain; ^6^ National Centre for the Treatment of Drug Addiction in Ljubljana Ljubljana Slovenia; ^7^ CONCEPT Center for Addiction‐Medicine Munich Germany; ^8^ Addiction Treatment Centre ‐ Ser.D ASL BI ‐ Local Health Unit Biella Italy; ^9^ Kantar Health New York New York; ^10^ The Kirby Institute UNSW Sydney Sydney Australia

**Keywords:** barriers, DAA, hepatitis C, opioid substitution therapy, OST, people who inject drugs, treatment

## Abstract

The aim of this analysis was to evaluate perceived barriers related to HCV testing, management and treatment among physicians practicing in clinics offering opioid agonist treatment (OAT). C‐SCOPE was a study consisting of a self‐administered survey among physicians practicing at clinics providing OAT in Australia, Canada, Europe and the United States between April and May 2017. A 5‐point Likert scale (1 = not a barrier, 3 = moderate barrier, 5 = extreme barrier) was used to measure responses to perceived barriers for HCV testing, evaluation and treatment across the domains of the health system, clinic and patient. Among the 203 physicians enrolled (40% USA, 45% Europe, 14% Australia/Canada), 21% were addiction medicine specialists, 29% psychiatrists and 69% were metro/urban. OAT physicians in this study reported poor access to on‐site venepuncture (35%), point‐of‐care HCV testing (16%), and noninvasive liver disease assessment (25%). Only 30% of OAT physicians reported personally treating HCV infection. Major perceived health system barriers to HCV management included the lack of funding for noninvasive liver disease testing, long wait times to see an HCV specialist, lack of funding for new HCV therapies, and reimbursement restrictions based on drug/alcohol use. Major perceived clinic barriers included the lack of peer support programmes and/or HCV case managers to facilitate linkage to care, the need to refer people off‐site for noninvasive liver disease staging, the lack of support for on‐site phlebotomy and the lack of on‐site delivery of HCV therapy. This study highlights several important modifiable barriers to enhance HCV testing, evaluation and treatment among PWID attending OAT clinics.

**Abbreviations**
APRI: AST to platelet ratio
DAA: direct‐acting antiviral
FIB‐4: fibrosis‐4
HCV: hepatitis C virus
OAT: opioid agonist treatment
OST: opioid substitution therapy
PWID: people who inject drugs

## 1 INTRODUCTION

The global burden of hepatitis C virus (HCV) infection is significant, with over 71 million people living with HCV 1 and 6.1 million people with recent injecting drug use.^2^ There is also a considerable burden of HCV among people with a history of injecting drug use and people receiving opioid agonist treatment (OAT) for opioid dependence.

High treatment completion and response to HCV therapy has been observed in people receiving OAT and people with recent injecting drug use.^3^ The integration and co‐location of care for HCV infection and OAT is associated with improved testing, linkage to treatment and retention in HCV care.^4^, ^5^, ^6^, ^7^ The high HCV prevalence among people who inject drugs (PWID) attending OAT clinics makes this an ideal setting for targeted strategies to enhance HCV care. However, there are still health system, structural, social, patient‐level and provider‐level barriers that are preventing broad uptake of HCV therapy among people receiving OAT or people with recent injecting drug use.^8^, ^9^, ^10^

In some countries, people receiving OAT and people with recent drug use are still ineligible 11 or considered unsuitable by practitioners 12 to receive direct‐acting antivirals (DAAs) , due to concerns of poor adherence, ongoing substance use, lower responses to therapy, medication price and the risk of reinfection.^9^, ^12^ In studies of general practitioners, a lack of confidence in initiating interferon‐based HCV treatment appears to have driven the low HCV screening, evaluation and treatment rates among this provider group.^13^ Low case numbers and inadequate HCV knowledge are important factors, with one study suggesting that primary care providers tend to underestimate efficacy and tolerability, and overestimate duration of DAAs,^14^ although many report a desire for more HCV education.^15^ Although qualitative interviews with providers have identified barriers to HCV care,^16^, ^17^ there are very few studies that have quantified potential modifiable barriers among physicians prescribing OAT.

The C‐SCOPE study was an international cross‐sectional study to evaluate clinic procedures and services, barriers, competency and attitudes towards HCV care among physicians practicing at clinics providing OAT.^18^ The aim of this analysis was to evaluate perceived barriers related to HCV testing, management and treatment among physicians prescribing OAT in the C‐SCOPE study.

## 2 METHODS

### 2.1 Study design, setting and participants

C‐SCOPE was an international cross‐sectional study that recruited physicians practicing at clinics providing OAT from Australia, Canada, Europe (Belgium, France, Germany, Italy, Portugal, Netherlands, Spain, Sweden and the United Kingdom) and the United States (US) from 14 April 2017 to 22 May 2017.^18^

Inclusion/exclusion criteria have been described in detail previously.^18^ Physicians must have spent at least 50% of time in clinics providing OAT treating patients or in management responsibilities, a minimum of 2 years treating patients in a clinic providing OAT, currently treating PWID with OAT, and working at a clinic, centre, department, or institution that is providing OAT and have been personally certified or allowed to prescribe OAT (Australia, Portugal and the United States only). Physicians working at the same clinic, centre, department or institution as two previous qualified respondents and those unwilling to comply with the study protocol were excluded.

Physicians were invited to participate via opt‐in online web panels (M3 Global Research panel), research databases and/or public and proprietary lists of clinics providing OAT in each country. The M3 Global Research panel is an actively managed double opt‐in online panel, for which physicians firstly opt‐in via an initial recruitment form and are then sent an automated email to confirm that they want to join the panel. Upon agreement to join the panel, M3 Global Research has a stringent verification process in order to confirm a respondent’s practicing status. In the United States, 100% of panellists are verified using the American Medical Association database.

Once identified, physicians were contacted via email or telephone and screened against the inclusion/exclusion criteria. Eligible physicians were invited to participate in an online survey in their local language. Physicians were provided an email and link to an online internet‐based survey. Approximately 2‐4 days after the initial invitation, people who did not initially respond were sent an e‐mail reminder. All participants gave written informed consent before study procedures started. Participating physicians were compensated for participation according to fair market value (from $90 to $165, depending on the country). Pearl IRB has determined this study meets the exemption requirements under 45CFR46.101(b).^2^

### 2.2 Study assessments

As previously described,^18^ physicians completed a survey to assess perceptions, self‐reported competency, and barriers related to the testing, management and treatment of HCV infection among those being prescribed OAT. Where applicable, questions were adapted from previously published studies.^9^, ^19^, ^20^, ^21^, ^22^, ^23^, ^24^

The survey included information on physician characteristics including region (Australia, Canada, Europe and the United States), primary specialty, and number of years in practice, practice source of funding (public, private for profit, private not for profit), type of OAT institution (substance use clinic/centre, hospital department that treats people on OAT, OAT clinic/centre and other institution/office that treats people on OAT), proportion of OAT therapy offered, OAT clinic setting (major metropolitan area, urban, suburb of large city, small city and rural/small town), number of patients managed on OAT who are PWID in past 12 months, number of patients personally managed on OAT who are PWID with HCV in the past 12 months, and training received. The survey also included information on OAT clinic characteristics including composition of teams that support OAT within the clinic, availability of HCV training for staff, existence of protocols and guidelines put in place, availability and location (on‐site, affiliated off‐site, not affiliated off‐site) of HCV diagnostic services, availability and location of HCV assessments prior to or during treatment, support services offered to HCV patients, use of electronic health records. Lastly, the survey included information on perceptions of barriers to HCV screening, testing and treatment (health system‐, clinic‐ and patients‐related barriers) and attitudes and perceptions towards HCV management.

### 2.3 Study outcomes and analysis

The availability of services for HCV testing and treatment at clinics among physicians who prescribe OAT were evaluated. Study endpoints also included physician‐perceived barriers regarding HCV testing, management and treatment of HCV for PWID. Physicians were asked to evaluate potential barriers for OAT patients to entering pathways to HCV care (ie testing and diagnosis), and to evaluate potential barriers to continuing pathways to HCV care (ie HCV treatment management). A 5‐point scale (1 = Not a barrier, 2 = Minor barrier, 3 = Moderate barrier, 4 = Major barrier and 5 = Extreme barrier) was used to measure barriers related to HCV testing, management and treatment. Barriers were categorized into those reporting greater than or equal to moderate barriers (eg moderate, major or extreme barriers). Analysis was performed using SAS 9.2 software (SAS Institute Inc., Cary, NC, USA).

## 3 RESULTS

### 3.1 Participant characteristics

Among 660 physicians contacted for this study, 203 physicians were enrolled (Table 1). Among the 457 who did not enrol in the study, 266 did not meet the inclusion criteria; 91 started the survey and did not complete it; and 100 were ‘over quota’ (by the time they responded to the survey, the quota for the target sample size for their country had already been met). The characteristics of OAT prescribers included in this study are highlighted in Table 1.

**Table 1:** Enrolment characteristics of physicians in the C‐SCOPE study (n = 203)

Variables	Overall n (%)
Region	
Europe	92 (45%)
United States	82 (40%)
Canada	16 (8%)
Australia	13 (6%)
Primary specialty of physician	
Psychiatry	58 (29%)
Addiction Medicine	43 (21%)
Addiction Psychiatry	40 (20%)
General Practice/Family Medicine	39 (19%)
Internal Medicine	14 (7%)
Neurology	6 (3%)
Other physician specialty	3 (1%)
Number of years in practice	
Mean (SD)	11 (8)
Median (Q1, Q3)	10 (5, 15)
Type of funding	
Public	108 (53%)
Private, for profit	60 (30%)
Private, not for profit	35 (17%)
Type of OAT institution	
Substance use clinic/centre	77 (38%)
Hospital department that treats people on OAT	42 (21%)
Opioid agonist therapy clinic/centre	31 (15%)
Other institution/office that treats people on OAT	54 (27%)
Per cent of patients receiving OAT [mean (SD)]	
Methadone	42% (35)
Buprenorphine	47% (35)
Heroin or diacetyl‐morphine	4% (10)
Other OAT	7% (18)
Setting of OAT clinic	
Major metropolitan area, population >500 000	82 (40%)
Urban area, population between 100 000 and 500 000	59 (29%)
Suburb of a large city, population >100 000	26 (13%)
Small city, population between 30 000 and 100 000	27 (13%)
Rural or small town, population <30 000	9 (4%)
Number of patients personally managed on OAT who are PWID	
Mean (SD)	51 (101)
Median (Q1, Q3)	20 (6, 50)
Number of patients personally managed who are PWID with HCV	
Mean (SD)	24 (50)
Median (Q1, Q3)	10 (2, 30)
Are you aware of any documents/tools for the screening, diagnosis or treatment of HCV?	
Yes	148 (73%)
No	55 (27%)
Have you obtained any information on screening, diagnosis or treatment of HCV infection in the past year?	
Yes	131 (65%)
No	72 (35%)
Have you attended training on HCV in the past year?	
Yes	75 (37%)
No	128 (63%)
Have you read or consulted the AASLD/IDSA, EASL or any other country specific guidelines?	
Yes	76 (37%)
No	127 (63%)

*Note*

Percentages indicate column percentages; OAT, opioid agonist therapy; SD, standard deviation; Q1, first quartile; Q3, third, quartile.

### 3.2 Existing team members to support agonist therapy within clinics providing OAT

The majority of physicians reported having nurses, nurse practitioners and medical assistants (71%), addiction medicine specialists (70%), psychiatrists (61%), primary care physicians (56%) and social workers (55%) as part of the team providing support for OAT at their clinic (Figure S1). Only 27% of clinics had a HCV specialist (Infectious Diseases, Hepatology or Gastroenterology). Very few clinics had a link‐to‐care coordinator (20%), peer support worker (16%) or HCV educator (10%).

### 3.3 Availability of HCV diagnostic services

**Figure 1:**
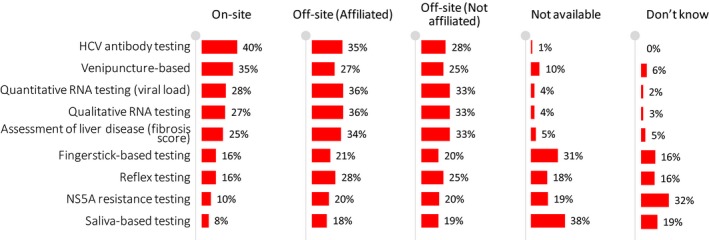
Availability of diagnostic services in clinics offering OAT

Physicians reported a wide range of diagnostic services being available to OAT providers, either on‐ or off‐site, with HCV testing (antibody and RNA) and noninvasive liver disease assessment being almost universally available (Figure 1 and Table S1). Only a minority of physicians reported having access to on‐site HCV antibody testing (40%), on‐site venipuncture (35%), quantitative (28%) or qualitative (27%) HCV RNA testing, or liver disease assessment (25%). In cases where HCV testing is performed off‐site, 97% of the clinics received the HCV test results from the same location where the tests are performed indicating that patients need to return to their OAT clinic to receive their results. On‐site availability of point‐of‐care fingerstick‐based (16%) and saliva‐based (8%) testing was uncommon. A large proportion of physicians did not have access to point‐of‐care finger‐stick‐based (31%) or saliva‐based (38%) testing.

### 3.4 Availability of testing protocols

A majority of physicians reported that their clinic has a protocol for HCV screening and diagnosis (n = 75, 37%) or that published guidelines are followed (n = 87, 43%; data not shown). From those 162 physicians, 86% reported testing all patients with history of injection drug use and 82% reported that HCV‐positive people are referred for additional testing and assessment. However, only 56% of physicians reported testing all OAT patients for HCV at first visit, 58% of physicians reported that patients are re‐tested on a regular basis, and 57% of physicians reported that patients receive test results and care referrals in one visit (Table S2).

### 3.5 Availability of electronic health records

Access to an electronic health record system was reported by 74% of physicians (n = 150), with: 40% of these reporting having electronic alerts for patients who are eligible/need testing/re‐testing; 30% reported that one of the elements of the system includes the generation of reports for those who were tested and the names of those who were HCV positive; 23% reporting that the system tracks, reports and facilitates reimbursement for HCV tests; and 14% report that the system provides an alert that patient is eligible for link‐to‐care services. Nearly half (42%) of the physicians did not report any of the above elements for the electronic health record system implemented in the clinic where they practice.

### 3.6 Availability of support services offered for HCV testing and diagnosis

The most common support service offered to enhance testing/diagnosis was appointment scheduling for HCV specialist (75% of physicians offered this service at their clinic) and informational posters to educate patients on prevalence, risk factors and recommendations (71%). The least common support service was patient financial incentives to attend HCV specialist appointments for testing/diagnosis (12%) (Table S3).

### 3.7 Availability of assessments prior to HCV treatment

Physicians reported a wide range of pre‐treatment assessments being available in OAT clinics (Figure S2). Standard HCV evaluation blood work was almost universally available. Although nearly half of physicians reported that their clinics have access to assessments such as creatinine (47%) or complete blood count (46%) available on‐site, the majority of patients had to be referred off‐site. However, only a minority of physicians reported having access to on‐site noninvasive liver fibrosis assessment, including AST to Platelet Ratio (APRI) (24%), Fibroscan (14%) or Fibrosis‐4 (FIB‐4) Index (11%).

### 3.8 Availability of services for HCV treatment

Only 30% of the OAT physicians in this study reported that they personally treat HCV infection. Thirty‐two per cent of physicians reported that they refer patients to HCV specialists in the same clinic and 62% reported that they refer HCV patients to other institutions for treatment. Overall, 26% of physicians reported that medication is dispensed at the OAT clinic, but most often, HCV medication is dispensed off‐site.

**Figure 2:**
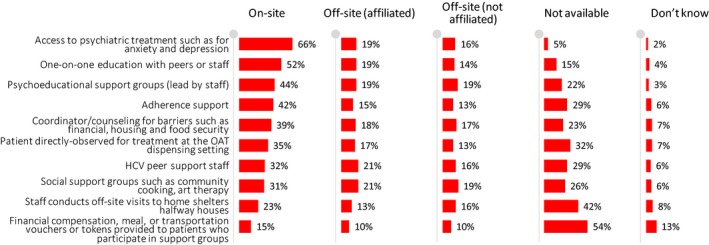
Availability of support services offered for HCV

The most common support services offered on‐site to enhance HCV treatment included access to psychiatric treatment (66%) and one‐on‐one education with peers/staff (52%) (Figure 2 and Table S4). Other common support services offered on‐site included psychoeducational support groups (44%), adherence support (42%), coordinator/counsellor for barriers such as financial, housing and food security (39%), directly observed therapy (35%) and HCV peer support (32%).

### 3.9 Physician perceived barriers to HCV screening and testing

**Figure 3:**
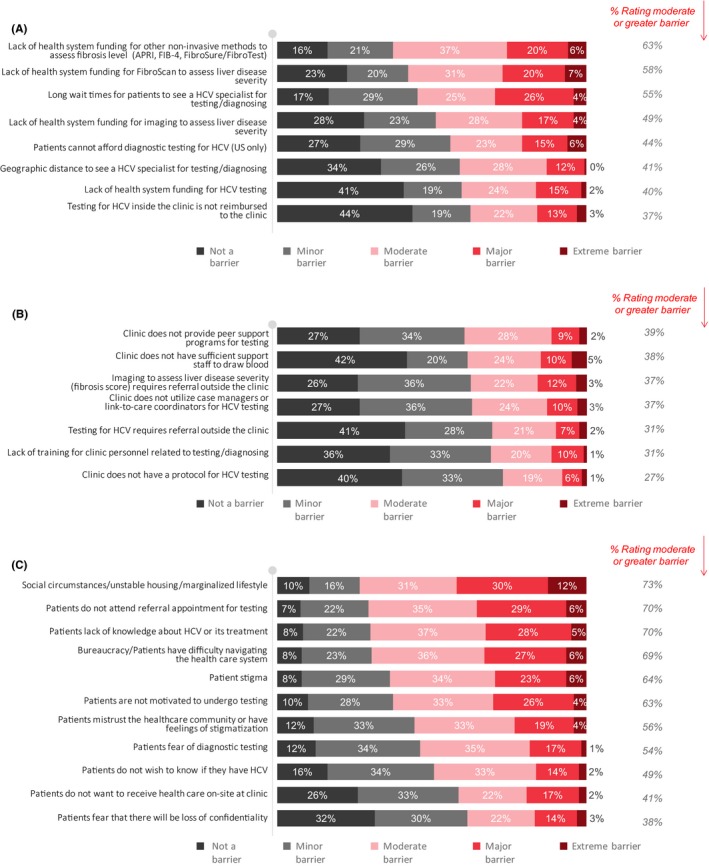
Perceived (A) health system‐related, (B) clinic‐related and (C) patient‐related barriers to HCV screening and testing among physicians prescribing opioid agonist treatment

At the level of the health system, the most common perceived barriers to HCV screening/testing by OAT physicians included a lack of health system funding for noninvasive methods of liver disease screening (63% ≥moderate; Figure 3A, Table S5). Other common barriers included long wait times for patients to see an HCV specialist (55% ≥moderate), patients not being able to afford diagnostic testing (the United States only) (44% ≥moderate), geographic distance to see an HCV specialist (41% ≥moderate) and lack of health system funding for HCV testing (40% ≥moderate).

At the level of the clinic, the most common perceived barriers to HCV screening and testing by OAT physicians included the lack of peer support programmes for testing (39% ≥moderate), the lack of support staff to draw blood (38% ≥moderate), the requirement for imaging outside the clinic (37% ≥moderate) and the fact that the clinic does not utilize case managers or link‐to‐care coordinators for HCV testing (37% ≥moderate; Figure 3B, Table S5).

At the level of the patient, the most common perceived barriers to HCV screening and testing by OAT physicians included social circumstances/unstable housing/marginalized lifestyle (73% ≥moderate), patients not attending appointments (70% ≥moderate), lack of knowledge of HCV and its treatment (70% ≥moderate), general difficulty navigating through the system (69% ≥moderate), patient stigma (64% ≥moderate) and concerns with patient motivation to get tested (63% ≥moderate) (Figure 3C, Table S5).

Other reported barriers included a lack of time for HCV testing due to competing responsibilities (47% ≥moderate) and that visits are too short and do not allow enough time for testing (41% ≥moderate) (Table S5).

### 3.10 Physician perceived barriers for HCV treatment

**Figure 4:**
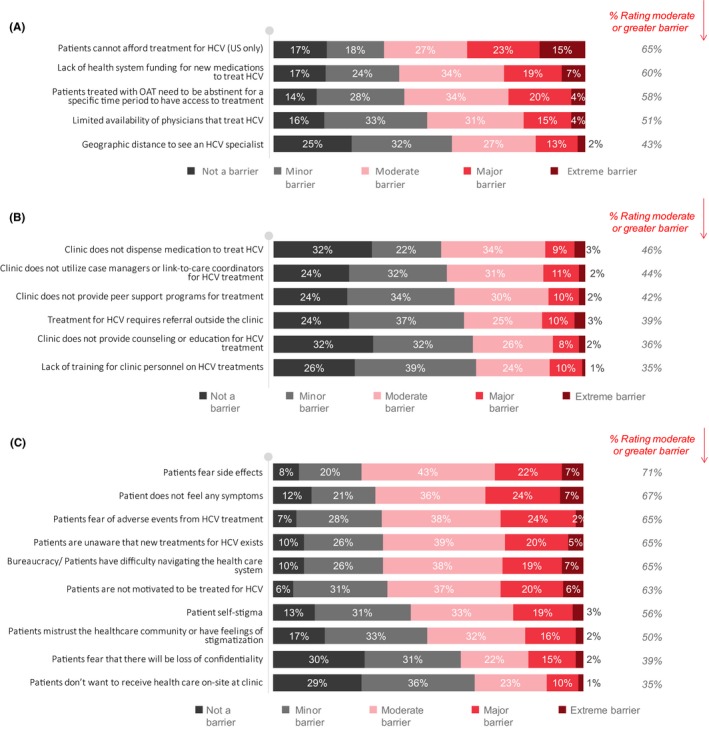
Perceived (A) health system‐related, (B) clinic‐related and (C) patient‐related barriers to HCV treatment among physicians

At the level of the health system, the most common perceived barriers for HCV treatment by OAT physicians are that the patients cannot afford HCV treatment (65% ≥moderate, the United States only), the lack of health system funding for new HCV medications to treat HCV (60% ≥moderate) and the requirement for a period of abstinence from recent drug use for government reimbursement of HCV therapy (58% ≥moderate) (Figure 4A, Table S6).

At the level of the clinic, the most common perceived barriers to HCV treatment by OAT physicians are the lack of delivery of therapy on‐site (46% ≥moderate), case managers (44% ≥moderate) and of peer support programmes for treatment (42% ≥moderate) (Figure 4B, Table S6).

The most common barriers for patients to continue the pathway to HCV care are probably, according to physicians, the patients’ fear of side effects and adverse events (71% ≥moderate and 65% ≥moderate), the asymptomatic nature of the infection (67% ≥moderate), patients being unaware that new treatments for HCV exist (65% ≥moderate), difficulty in navigating the healthcare system (65% ≥moderate), concerns around patient motivation to be treated for HCV (63% ≥moderate), stigma experienced by patients (56% ≥moderate) and mistrust of the healthcare system (50% ≥moderate) (Figure 4C, Table S6).

### 3.11 Physician attitudes towards HCV treatment in people receiving OST

**Figure 5:**
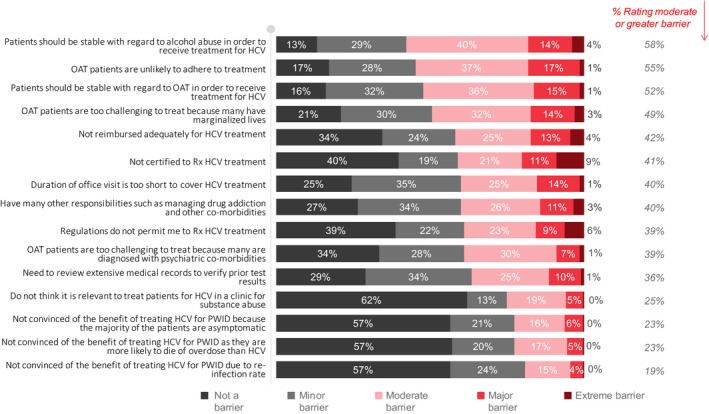
Physician attitudes towards perceived barriers to HCV treatment

Physicians were asked a variety of questions about HCV treatment in people receiving OAT. The most common barriers identified included the perceived need for stable alcohol use (58% ≥moderate), concerns of adherence (55% ≥moderate), requirement for stable OAT (52% ≥moderate) and the challenging and marginalized lives of patients (49% ≥moderate). Only 25% reported that treating patients for HCV infection in a clinic for substance use was a ≥moderate barrier (Figure 5, Table S7).

## 4 DISCUSSION

This study describes physician perceived barriers related to testing and treatment of HCV infection in an international sample of physicians prescribing OAT. OAT physicians in this study reported poor access to strategies that have been demonstrated to improve HCV testing and linkage to care, such as on‐site HCV testing, point‐of‐care HCV testing, noninvasive liver disease assessment and electronic prompts for HCV testing. Further, less than one‐third of OAT physicians reported personally treating HCV infection, with the majority referring HCV patients off‐site for treatment. This study also highlighted the most common perceived barriers to testing and treatment for HCV infection among OAT prescribers at the level of the system, clinic and patient. To our knowledge, this is one of the first studies to comprehensively evaluate the relative importance of potential modifiable barriers to enhance HCV care in drug treatment settings among an international sample of physicians who prescribe OAT.

The minority of OAT physicians in the C‐SCOPE study reported having access to on‐site HCV testing and liver disease assessment. Current testing algorithms involve detection of HCV antibodies to confirm exposure, followed by HCV RNA testing to detect active infection. This two‐step pathway requires up to 5 visits to practitioners and off‐site phlebotomists, leading to a drop‐off in those receiving a diagnosis of active infection.^25^ In studies from Australia, Canada, and the United States, among people testing anti‐HCV antibody positive, only 46‐73% of people received confirmatory HCV RNA testing.^26^, ^27^, ^28^, ^29^, ^30^, ^31^ On‐site testing, point‐of‐care testing, dried‐blood spot testing and noninvasive liver disease assessment have also been shown to be effective to increase uptake of HCV testing 7, 32, 33, 34 and linkage to HCV care.^33^ On‐site testing and care has the potential to reduce non‐attendance to off‐site phlebotomy, provides more immediate results to facilitate enhanced counselling, education and linkage to care, and provides a more effective referral mechanism (via on‐site care) as compared to referring patients off‐site.^10^, ^16^, ^17^, ^35^, ^36^, ^37^, ^38^ In fact, the recent availability of finger‐stick HCV RNA testing with good diagnostic accuracy may further provide opportunity for exploring opportunities for on‐site diagnosis and linkage to treatment in a single‐visit.^39^, ^40^, ^41^ Further efforts are needed to integrate and scale‐up HCV testing and linkage to treatment within drug treatment clinics.

It is concerning that only a minority of OAT physicians reported having protocols in their clinics or follow published guidelines for HCV testing. In previous work from the C‐SCOPE study among OAT providers, competency with respect to ensuring that people at‐risk of HCV regularly screened self‐reported as less than average was shown to be associated with a lack of awareness of documents/tools for the screening, diagnosis or treatment of HCV; not having obtained information on screening, diagnosing or treatment of HCV; not having attended any training on HCV in the past year, and no protocol in place for HCV testing.^18^ As such, strategies are required to improve HCV education and training about HCV testing and diagnosis among physicians practicing in clinics offering OAT.

Despite 74% of OAT physicians reporting access to an electronic health record, nearly half reported that they did not have elements which would enable them to create electronic alerts for patients who require testing or re‐testing. Clinician reminders to prompt HCV testing during clinical visits have been associated with increased HCV testing rates.^6^ Innovative strategies to utilize existing electronic health records to facilitate enhanced screening and testing in drug treatment clinics should be explored.

At the level of the health system, major perceived barriers to HCV testing and treatment by OAT prescribers in the C‐SCOPE study included the lack of funding/access for diagnostic testing, for noninvasive liver disease screening and for DAA treatment, as well as the requirement in some settings for a period of abstinence from drug use in order to be eligible for the government reimbursement of HCV therapy. Restrictions set by payers, including national governments and others, in response to the initially high list prices of DAA therapies have been documented in a number of different jurisdictions.^11^, ^42^, ^43^ However, many governments/payers internally have begun to remove restrictions based on liver disease and recent drug use for the reimbursement of DAA therapies.^43^ Nevertheless, further work is needed to address these systematic barriers to access for diagnostics and treatment. Given that HCV treatment is effective in people receiving OAT and people with ongoing drug use (either injecting or noninjecting),^3^ further work is also needed to ensure the removal of restrictions for the reimbursement of DAA therapy based on recent drug use.

At the level of the clinic, a lack of peer support programmes and/or HCV case managers to facilitate linkage to care was noted by OAT prescribers. There is evidence demonstrating the value of peer‐based HCV support programmes and HCV case managers for improving engagement in HCV testing and treatment.^44^, ^45^, ^46^, ^48^ In a recent, randomized controlled trial, 144 people living with HCV‐HIV co‐infection and with substance use disorders were randomized to three treatment groups including usual care (n = 36), usual care plus cash incentives (n = 54) and usual care plus peer mentors (n = 54) to evaluate the rate of HCV treatment uptake and cure. Overall, the treatment uptake was higher in people randomized to peers (83%) or cash (76%, 41 of 54) compared to usual care (67%, 24 of 36), although this was not statistically significant (*P* = 0.11). These results are encouraging, but further work is needed to evaluate peer‐based strategies to enhance HCV testing and treatment uptake.

At the level of the clinic, major perceived barriers to HCV testing and treatment by OAT prescribers in the C‐SCOPE study included the need to refer people off‐site for noninvasive liver disease staging, the lack of support for on‐site phlebotomy and the lack of delivery of HCV therapy on‐site. Given that both patients and providers report on‐site testing to be preferable to referral to off‐site phlebotomists and tertiary clinics 16, 17, 36, 37, 38 and that on‐site testing (including point‐of‐care HCV testing) can improve linkage to HCV testing 7, 32, 33, 34 and care,^33^ strategies to enhance access to simple on‐site HCV and liver disease testing should be implemented. It is interesting that a large proportion of participants reported a lack of access to noninvasive fibrosis assessments (eg APRI and FIB‐4) that can be calculated from standard HCV blood work, suggesting that further education is needed about the potential utility of these tests to assess liver disease. The co‐location of care for HCV infection and OAT is associated with improved uptake of HCV testing and treatment, and improved retention in HCV care.^4^, ^5^, ^6^, ^7^ As such, strategies to enhance the co‐location of services for HCV care and OAT treatment should be explored given their potential to improve engagement in HCV testing and management.

At the level of the patient, the most common perceived barriers to HCV screening and testing by OAT physicians included social circumstances (eg unstable housing), lack of attendance to appointments, poor knowledge of HCV and its treatment (including awareness of new treatments), fear of treatment side effects, the asymptomatic nature of HCV infection, difficulties navigating through the health system, stigma and mistrust of the healthcare system. These barriers reported by OAT prescribers are consistent with epidemiological studies and surveys of patients who inject drugs living with HCV infection,^9^, ^10^, ^17^, ^37^, ^38^, ^49^ albeit most studies have been in the interferon‐era. It is important to note that the relative ranking of these barriers is based on the perceptions of OAT providers in the C‐SCOPE study, not the opinions of people living with HCV infection. Further research is needed to better understand barriers to HCV care among people who inject drugs in the DAA era.

Several limitations must be considered in the interpretation of the current results. Study participants were recruited through opt‐in online web panels, research databases and/or public and proprietary lists of clinics providing OAT in each country, and thus possible selection bias may exist among those who chose to participate. However, it should be noted that the membership of the US M3 Global Research panel closely matches the demographics of the American Medical Association statistics with respect to region, age and gender, which provides some confidence in the observed results. The survey data were also based on self‐report and thus subject to recall bias. Finally, physicians prescribing OAT were recruited across several different geographical regions and countries and thus there exists heterogeneity in the availability of healthcare services, education and training, and policies for HCV testing and treatment.

Irrespective of these limitations, to our knowledge, this is one of the first studies to systematically evaluate perceived barriers related to testing, management and treatment of HCV infection among an international sample of physicians prescribing OAT. This study highlights important modifiable barriers to enhance HCV care in drug treatment settings. Given the growing body of evidence of the effectiveness of different interventions to enhance HCV testing, linkage to care and treatment,^6^, ^7^ including among PWID,^7^ it is critical to explore strategies to address these barriers. Moving forward, the challenge will be tailoring successful interventions to the particular jurisdiction and/or setting, given that models of care will differ and one size will not fit all.^23^

## CONFLICT OF INTEREST

JG is a consultant/advisor and has received research grants from AbbVie, Cepheid, Gilead Sciences and Merck Sharp & Dohme Corp., a subsidiary of Merck & Co., Inc., Kenilworth, NJ, USA. MT is a consultant/advisor for Gilead Sciences and Merck Sharp & Dohme Corp. AK is a consultant/advisor and/or speaker for Alkaloid, Carso, Eli Lilly, Gillead Sciences, Medis/Mundipharma and Merck Sharp & Dohme Corp., a subsidiary of Merck & Co. SW is consultant/advisor for AbbVie, MSD/Merck, Gilead. EM, JE and RL are employees of Kantar Health, paid consultants of Merck Sharp & Dohme Corp., a subsidiary of Merck & Co., Inc., Kenilworth, NJ, USA. AL is a consultant/advisor and has received research grants from AbbVie, Gilead Sciences and Merck Pharmaceuticals. JG, MT, AK, SW, LS and AL were consultants/advisors for this study.
