# Supplementary material for: Perceived barriers related to testing, management and treatment of HCV infection among physicians prescribing opioid agonist therapy: The C‐SCOPE Study
Source: J Viral Hepat. 2019 Jun 11;26(9):1094–104. doi: 10.1111/jvh.13119 (PMC6771477; doi:10.1111/jvh.13119)
Supplement: Supplementary file 1 [file JVH-26-1094-s001.docx]

**SUPPLEMENTARY MATERIAL**

Supplementary Figure 1. Individuals part of the multidisciplinary team to support OAT care (n=203)


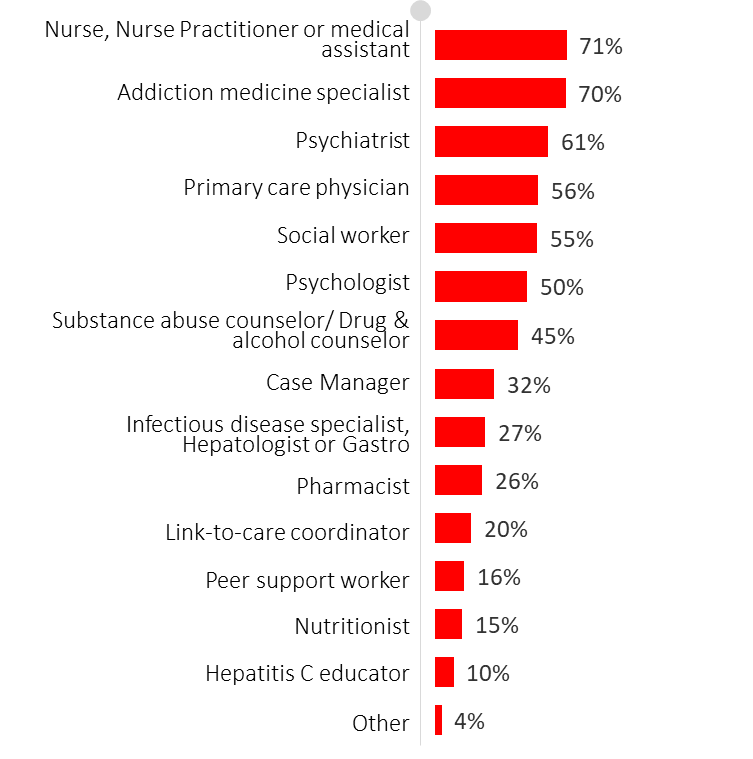


**Supplementary Figure 2. Availability of HCV assessments prior to or during HCV treatment (n=203)
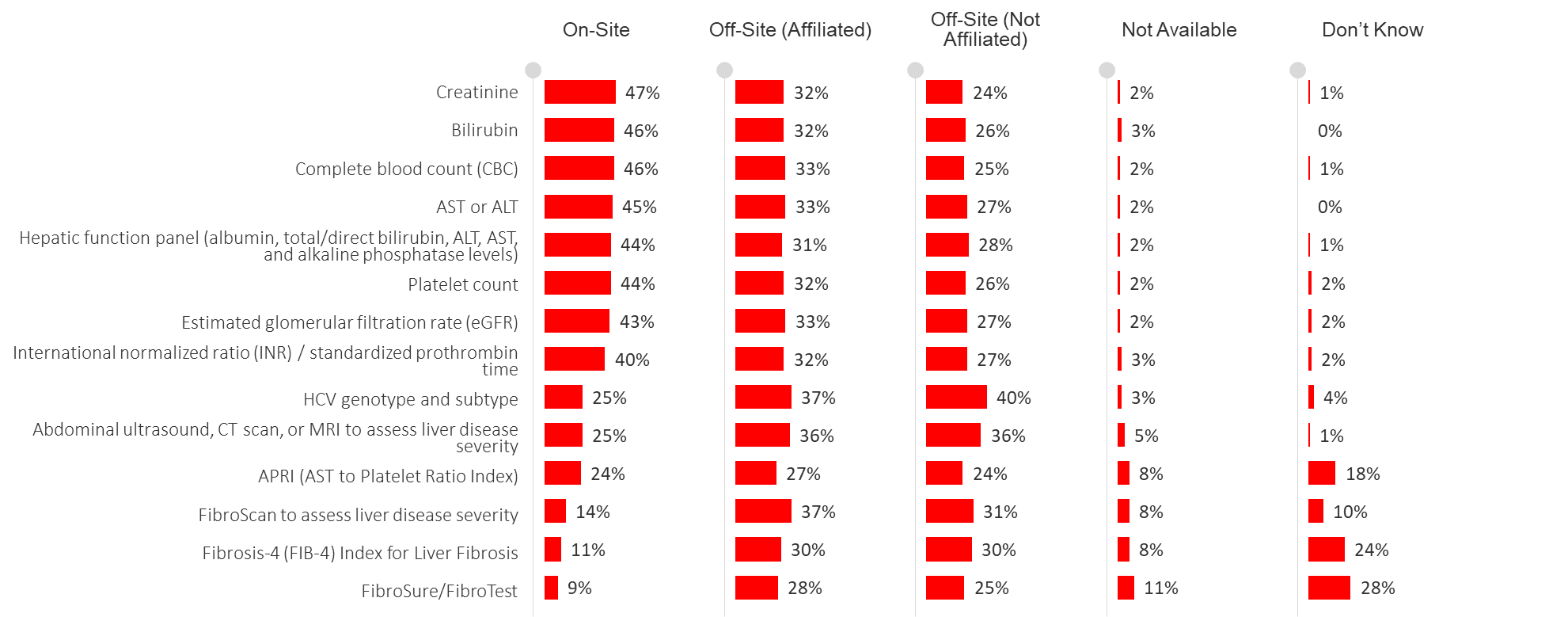
**

**Supplementary Table 1. Availability of diagnostic services in clinics offering OAT (n=203)**

|  | **On-site n (%)** | **Off-site (Affiliated)  n (%)** | **Off-site**  **(Not Affiliated)  n (%)** | **Not Available  n (%)** | **Do not know  n (%)** |
| --- | --- | --- | --- | --- | --- |
| HCV antibody testing | 82 (40%) | 71 (35%) | 57 (28%) | 3 (1%) | 0 (0%) |
| Venipuncture-based HCV testing | 72 (35%) | 54 (27%) | 51 (25%) | 20 (10%) | 13 (6%) |
| Quantitative HCV-RNA testing (viral load) | 56 (28%) | 73 (36%) | 67 (33%) | 8 (4%) | 5 (2%) |
| Qualitative HCV-RNA testing | 55 (27%) | 73 (36%) | 66 (33%) | 8 (4%) | 6 (3%) |
| Assessment of liver disease (fibrosis score) | 50 (25%) | 69 (34%) | 67 (33%) | 11 (5%) | 11 (5%) |
| Fingerstick-based HCV testing | 33 (16%) | 43 (21%) | 40 (20%) | 62 (31%) | 33 (16%) |
| Reflex testing | 33 (16%) | 56 (28%) | 51 (25%) | 37 (18%) | 32 (16%) |
| NS5A resistance testing | 21 (10%) | 40 (20%) | 41 (20%) | 39 (19%) | 65 (32%) |
| Saliva-based HCV testing | 16 (8%) | 36 (18%) | 39 (19%) | 78 (38%) | 38 (19%) |

**Supplementary Table 2. Features included in institution’s Protocol/Guidelines for screening and diagnosing HCV among participants with a protocol or following guidelines at their clinic (n=162)**

|  | **Yes n (%)** | **No  n (%)** | **Do not know  n (%)** |
| --- | --- | --- | --- |
| Clinic tests all patients with injection-drug use (current or ever, including those who injected once) for HCV | 140 (86%) | 21 (13%) | 1 (1%) |
| Patients testing positive for HCV are referred to a HCV treating physician for additional testing and assessment | 133 (82%) | 27 (17%) | 2 (1%) |
| HCV testing protocol includes HIV testing | 126 (78%) | 29 (18%) | 7 (4%) |
| Eligible patients are notified that they would be tested for HCV unless they opt out | 118 (73%) | 40 (25%) | 4 (2%) |
| Clinic tests all patients at risk of reinfection after previous spontaneous clearance or treatment-related SVR | 113 (70%) | 36 (22%) | 13 (8%) |
| Nurse or other staff member guides patients through HCV testing process | 99 (61%) | 59 (36%) | 4 (2%) |
| Patients are re-tested on regular basis | 94 (58%) | 62 (38%) | 6 (4%) |
| Clinic tests all patients for HCV at first visit | 90 (56%) | 69 (43%) | 3 (2%) |
| Patients receive test results and care referrals in one visit | 93 (57%) | 66 (41%) | 3 (2%) |
| Nurse/medical assistant identifies and reminds physician with a chart note of patients who meet criteria for testing | 84 (52%) | 76 (47%) | 2 (1%) |
| Nurse/medical assistant initiates test | 83 (51%) | 74 (46%) | 5 (3%) |
| Clinic tests all patients born between 1945 and 1965 for HCV | 67 (41%) | 82 (51%) | 13 (8%) |

**Supplementary Table 3. The availability of support services offered for HCV testing and diagnosis in clinics offering OAT (n=203)**

|  | **Availablen (%)** | **Not Availablen (%)** | **Do not know  n (%)** |
| --- | --- | --- | --- |
| Appointment scheduling for HCV specialist for testing/diagnosing | 153 (75%) | 46 (23%) | 4 (2%) |
| Informational posters to educate patients on prevalence, risk factors and recommendations on HCV testing | 145 (71%) | 48 (24%) | 10 (5%) |
| Reminder phone calls for HCV specialist appointments for testing/diagnosing | 99 (49%) | 80 (39%) | 24 (12%) |
| Peer support offered on-site | 81 (40%) | 106 (52%) | 16 (8%) |
| Staff accompanies patient to off-site testing/diagnosing for HCV when necessary | 76 (37%) | 117 (58%) | 10 (5%) |
| Patient financial incentives to attend HCV specialist appointments for testing/diagnosing | 25 (12%) | 159 (78%) | 19 (9%) |
|  |  |  |  |
|  |  |  |  |
|  |  |  |  |
|  |  |  |  |

**Supplementary Table 4. The availability of support services offered for HCV treatment in clinics offering OAT (n=203)**

|  | **On-site n (%)** | **Off-site (Affiliated)  n (%)** | **Off-site**  **(Not Affiliated)  n (%)** | **Not Available  n (%)** | **Do not know  n (%)** |
| --- | --- | --- | --- | --- | --- |
| Access to psychiatric treatment such as for anxiety and depression | 134 (66%) | 39 (19%) | 32 (16%) | 10 (5%) | 5 (3%) |
| One-on-one education with peers or staff? | 106 (52%) | 38 (19%) | 29 (14%) | 30 (15%) | 8 (4%) |
| Psychoeducational support groups (lead by staff) | 89 (44%) | 38 (19%) | 38 (19%) | 44 (22%) | 7 (3%) |
| Adherence support including patient directly observed treatment through OAT dispensing setting, smartphone or text | 85 (42%) | 31 (15%) | 26 (13%) | 58 (29%) | 13 (6%) |
| Coordinator/counselling for barriers such as financial, housing and food security | 80 (39%) | 37 (18%) | 34 (17%) | 47 (23%) | 15 (7%) |
| Patient directly-observed for HCV treatment at the OAT dispensing setting | 72 (35%) | 35 (17%) | 26 (13%) | 65 (32%) | 15 (7%) |
| HCV peer support staff | 64 (32%) | 43 (21%) | 33 (16%) | 58 (29%) | 12 (6%) |
| Social support groups such as community cooking, art therapy | 63 (31%) | 43 (21%) | 39 (19%) | 52 (26%) | 13 (6%) |
| Staff conducts off-site visits to home shelters halfway houses | 47 (23%) | 27 (13%) | 33 (16%) | 85 (42%) | 17 (8%) |
| Financial compensation, meal, or transportation vouchers or tokens provided to patients who participate in support groups | 30 (15%) | 21 (10%) | 21 (10%) | 109 (54%) | 26 (13%) |

Supplementary Table 5: Perceived barriers to HCV screening and testing among physicians practicing in clinics offering OAT in the C-SCOPE study (n=203)

|  | **Extreme barrier n (%)** | **Major**  **barrier  n (%)** | **Moderate barrier  n (%)** | **Minor barrier  n (%)** | **Not a Barrier  n (%)** | **>Moderate Barrier**  **n (%)** |
| --- | --- | --- | --- | --- | --- | --- |
| ***Health system barriers*** |  |  |  |  |  |  |
| Lack of health system funding for other non-invasive methods to assess fibrosis (e.g. APRI, FIB-4, FibroSure/FibroTest) | 12 (6%) | 40 (20%) | 75 (37%) | 43 (21%) | 33 (16%) | 127 (63%) |
| Lack of health system funding for FibroScan to assess liver disease severity | 14 (7%) | 41 (20%) | 62 (31%) | 40 (20%) | 46 (23%) | 117 (58%) |
| Long wait times for patients to see an HCV specialist (from referral to assessment) for testing/diagnosing | 8 (4%) | 52 (26%) | 51 (25%) | 58 (29%) | 34 (17%) | 111 (55%) |
| Lack of health system funding for imaging to assess liver disease severity | 8 (4%) | 34 (17%) | 57 (28%) | 47 (23%) | 57 (28%) | 99 (49%) |
| Patients cannot afford diagnostic testing for hepatitis C (US Only, base n=82) | 5 (6%) | 12 (15%) | 19 (23%) | 24 (29%) | 22 (27%) | 36 (44%) |
| Geographic distance to see an HCV specialist for testing/diagnosing | 1 (0%) | 25 (12%) | 57 (28%) | 52 (26%) | 68 (34%) | 83 (41%) |
| Lack of health system funding for HCV testing | 3 (1%) | 30 (15%) | 49 (24%) | 38 (19%) | 83 (41%) | 82 (40%) |
| Testing for HCV inside the clinic is not reimbursed to the clinic | 6 (3%) | 26 (13%) | 44 (22%) | 38 (19%) | 89 (44%) | 76 (37%) |
|  |  |  |  |  |  |  |
|  |  |  |  |  |  |  |
| ***Clinic barriers*** |  |  |  |  |  |  |
| Clinic does not provide peer support programs for testing | 5 (2%) | 18 (9%) | 57 (28%) | 69 (34%) | 54 (27%) | 80 (39%) |
| Clinic does not have sufficient support staff to draw blood | 10 (5%) | 20 (10%) | 48 (24%) | 40 (20%) | 85 (42%) | 78 (38%) |
| Imaging to assess liver disease severity requires referral outside the clinic | 7 (3%) | 25 (12%) | 44 (22%) | 74 (36%) | 53 (26%) | 76 (37%) |
| Clinic does not utilize case managers or link-to-care coordinators for HCV testing | 6 (3%) | 20 (10%) | 49 (24%) | 74 (36%) | 54 (27%) | 75 (37%) |
| Testing for HCV requires referral outside the clinic | 5 (2%) | 15 (7%) | 42 (21%) | 57 (28%) | 84 (41%) | 62 (31%) |
| Lack of training for clinic personnel related to testing/diagnosing | 2 (1%) | 21 (10%) | 40 (20%) | 66 (33%) | 74 (37%) | 63 (31%) |
| Clinic does not have a protocol for HCV testing | 3 (1%) | 13 (6%) | 39 (19%) | 66 (33%) | 82 (40%) | 55 (27%) |
|  |  |  |  |  |  |  |
|  |  |  |  |  |  |  |
| ***Patient barriers*** |  |  |  |  |  |  |
| Social circumstances/unstable housing/marginalized lifestyle | 25 (12%) | 60 (30%) | 64 (31%) | 33 (16%) | 21 (10%) | 149 (73%) |
| Patients do not attend referral appointment for testing | 13 (6%) | 59 (29%) | 71 (35%) | 45 (22%) | 15 (7%) | 143 (70%) |
| Patients lack knowledge about HCV or its treatment | 10 (5%) | 56 (28%) | 76 (37%) | 44 (22%) | 17 (8%) | 142 (70%) |
| Bureaucracy/Patients have difficulty navigating the health care system | 13 (6%) | 54 (27%) | 74 (36%) | 46 (23%) | 16 (8%) | 141 (69%) |
| Patient stigma | 13 (6%) | 47 (23%) | 69 (34%) | 58 (29%) | 16 (8%) | 129 (64%) |
| Patients are not motivated to undergo testing | 8 (4%) | 53 (26%) | 66 (33%) | 56 (28%) | 20 (10%) | 127 (63%) |
| Patients mistrust the healthcare community or have feelings of stigmatization | 8 (4%) | 39 (19%) | 66 (33%) | 66 (33%) | 24 (12%) | 113 (56%) |
| Patients fear of diagnostic testing | 3 (1%) | 34 (17%) | 72 (35%) | 69 (34%) | 25 (12%) | 109 (54%) |
| Patients do not wish to know if they have HCV | 5 (2%) | 28 (14%) | 67 (33%) | 70 (34%) | 33 (16%) | 100 (49%) |
| Patients do not want to receive health care on-site at clinic | 5 (2%) | 34 (17%) | 45 (22%) | 66 (33%) | 53 (26%) | 84 (41%) |
| Patients fear that there will be loss of confidentiality | 6 (3%) | 28 (14%) | 44 (22%) | 61 (30%) | 64 (32%) | 78 (38%) |
|  |  |  |  |  |  |  |
|  |  |  |  |  |  |  |
| ***Other barriers*** |  |  |  |  |  |  |
| I have many other responsibilities such as managing drug addiction and other co-morbidities | 12 (6%) | 23 (11%) | 61 (30%) | 55 (27%) | 52 (26%) | 96 (47%) |
| Duration of office visit is too short to cover HCV testing | 4 (2%) | 22 (11%) | 58 (29%) | 59 (29%) | 60 (30%) | 84 (41%) |
| HCV testing is not an important function to be performed by on-site provider but should be done by primary care provider | 3 (1%) | 24 (12%) | 48 (24%) | 57 (28%) | 71 (35%) | 75 (37%) |
| I am not reimbursed adequately for HCV testing | 9 (4%) | 21 (10%) | 42 (21%) | 57 (28%) | 74 (36%) | 72 (35%) |

Supplementary Table 6: Perceived barriers to HCV treatment among physicians practicing in clinics offering OAT in the C-SCOPE study (n=203)

|  | **Extreme barrier n (%)** | **Major**  **barrier  n (%)** | **Moderate barrier  n (%)** | **Minor barrier  n (%)** | **Not a Barrier  n (%)** | **>Moderate Barrier**  **n (%)** |
| --- | --- | --- | --- | --- | --- | --- |
| ***Health system barriers*** |  |  |  |  |  |  |
| Patients cannot afford treatment for hepatitis C (US only, base n=82) | 12 (15%) | 19 (23%) | 22 (27%) | 15 (18%) | 14 (17%) | 53 (65%) |
| Lack of health system funding for new medications to treat hepatitis C | 15 (7%) | 38 (19%) | 68 (34%) | 48 (24%) | 34 (17%) | 121 (60%) |
| Patients treated with opioid agonist therapy need to be abstinent for a specific time period to have access to treatment | 9 (4%) | 41 (20%) | 68 (34%) | 57 (28%) | 28 (14%) | 118 (58%) |
| Limited availability of physicians that treat hepatitis C | 9 (4%) | 31 (15%) | 63 (31%) | 67 (33%) | 33 (16%) | 103 (51%) |
| Geographic distance to see a hepatitis C specialist | 5 (2%) | 27 (13%) | 55 (27%) | 65 (32%) | 51 (25%) | 87 (43%) |
|  |  |  |  |  |  |  |
| ***Clinic barriers*** |  |  |  |  |  |  |
| Clinic does not dispense medication to treat hepatitis C | 7 (3%) | 19 (9%) | 68 (34%) | 45 (22%) | 64 (32%) | 94 (46%) |
| Clinic does not utilize case managers or link-to-care coordinators for hepatitis C treatment | 4 (2%) | 23 (11%) | 63 (31%) | 64 (32%) | 49 (24%) | 90 (44%) |
| Clinic does not provide peer support programs for treatment | 4 (2%) | 20 (10%) | 61 (30%) | 70 (34%) | 48 (24%) | 85 (42%) |
| Treatment for hepatitis C requires referral outside the clinic | 7 (3%) | 21 (10%) | 51 (25%) | 75 (37%) | 49 (24%) | 79 (39%) |
| Clinic does not provide counseling or education for hepatitis C treatment | 4 (2%) | 17 (8%) | 52 (26%) | 65 (32%) | 65 (32%) | 73 (36%) |
| Lack of training for clinic personnel on hepatitis C treatments | 2 (1%) | 21 (10%) | 48 (24%) | 79 (39%) | 53 (26%) | 71 (35%) |
|  |  |  |  |  |  |  |
| ***Patient barriers*** |  |  |  |  |  |  |
| Patients fear side effects | 14 (7%) | 44 (22%) | 87 (43%) | 41 (20%) | 17 (8%) | 145 (71%) |
| Patient does not feel any symptoms | 15 (7%) | 49 (24%) | 72 (36%) | 42 (21%) | 25 (12%) | 136 (67%) |
| Patients fear of adverse events from hepatitis C treatment | 5 (2%) | 49 (24%) | 78 (38%) | 56 (28%) | 15 (7%) | 132 (65%) |
| Patients are unaware that new treatments for hepatitis C exists | 10 (5%) | 41 (20%) | 80 (39%) | 52 (26%) | 20 (10%) | 131 (65%) |
| Bureaucracy/Patients have difficulty navigating the health care system | 14 (7%) | 39 (19%) | 78 (38%) | 52 (26%) | 20 (10%) | 131 (65%) |
| Patients are not motivated to be treated for hepatitis C | 13 (6%) | 41 (20%) | 74 (37%) | 62 (31%) | 13 (6%) | 128 (63%) |
| Patient self-stigma | 7 (3%) | 39 (19%) | 67 (33%) | 63 (31%) | 27 (13%) | 113 (56%) |
| Patients mistrust the healthcare community or have feelings of stigmatization | 5 (2%) | 33 (16%) | 64 (32%) | 67 (33%) | 34 (17%) | 102 (50%) |
| Patients fear that there will be loss of confidentiality | 5 (2%) | 31 (15%) | 44 (22%) | 63 (31%) | 60 (30%) | 80 (39%) |
| Patients don’t want to receive health care on-site at clinic | 3 (1%) | 21 (10%) | 47 (23%) | 74 (36%) | 58 (29%) | 71 (35%) |

Supplementary Table 7: Physician attitudes towards perceived barriers to HCV treatment among physicians practicing in clinics offering OAT in the C-SCOPE study (n=203)

|  | **Extreme barrier n (%)** | **Major**  **barrier  n (%)** | **Moderate barrier  n (%)** | **Minor barrier  n (%)** | **Not a Barrier  n (%)** | **>Moderate Barrier**  **n (%)** |
| --- | --- | --- | --- | --- | --- | --- |
| Patients should be stable (adherent to treatment or recommended lifestyle changes) with regard to alcohol abuse in order to receive treatment for hepatitis C | 8 (4%) | 29 (14%) | 80 (40%) | 59 (29%) | 27 (13%) | 117 (58%) |
| Opioid agonist therapy patients are unlikely to adhere to treatment | 3 (1%) | 34 (17%) | 74 (37%) | 57 (28%) | 35 (17%) | 111 (55%) |
| Patients should be stable (adherent to treatment and/or recommended lifestyle changes) with regard to opioid agonist therapy in order to receive treatment for hepatitis C | 3 (1%) | 30 (15%) | 73 (36%) | 64 (32%) | 33 (16%) | 106 (52%) |
| Opioid agonist therapy patients are too challenging to treat because many have marginalized lives, making it difficult for them to keep appointments | 6 (3%) | 29 (14%) | 65 (32%) | 60 (30%) | 43 (21%) | 100 (49%) |
| I am not reimbursed adequately for HCV treatment | 9 (4%) | 26 (13%) | 50 (25%) | 48 (24%) | 70 (34%) | 85 (42%) |
| I am not certified to prescribe HCV treatment | 18 (9%) | 23 (11%) | 42 (21%) | 39 (19%) | 81 (40%) | 83 (41%) |
| Duration of office visit is too short to cover HCV treatment | 3 (1%) | 28 (14%) | 50 (25%) | 72 (35%) | 50 (25%) | 81 (40%) |
| I have many other responsibilities such as managing drug, alcohol and other co-morbidities | 7 (3%) | 22 (11%) | 52 (26%) | 68 (34%) | 54 (27%) | 81 (40%) |
| Regulations do not permit me to prescribe HCV treatment | 13 (6%) | 19 (9%) | 47 (23%) | 45 (22%) | 79 (39%) | 79 (39%) |
| Opioid agonist therapy patients are too challenging to treat because many are diagnosed with psychiatric co-morbidities, such as depression and anxiety | 3 (1%) | 15 (7%) | 61 (30%) | 56 (28%) | 68 (34%) | 79 (39%) |
| I need to review extensive medical records to verify prior test results | 2 (1%) | 21 (10%) | 51 (25%) | 70 (34%) | 59 (29%) | 74 (36%) |
| I do not think it is relevant to treat patients for hepatitis C in a clinic for substance abuse | 1 (0%) | 11 (5%) | 39 (19%) | 26 (13%) | 126 (62%) | 51 (25%) |
| I am not convinced of the benefit of treating HCV for people who inject drugs because the majority of the patients are asymptomatic or have low fibrosis score | 1 (0%) | 12 (6%) | 33 (16%) | 42 (21%) | 115 (57%) | 46 (23%) |
| I am not convinced of the benefit of treating HCV for people who inject drugs as they are more likely to die of overdose than HCV infection and its complications | 1 (0%) | 10 (5%) | 35 (17%) | 41 (20%) | 116 (57%) | 46 (23%) |
| I am not convinced of the benefit of treating HCV for PWID due to re-infection rate | 1 (0%) | 8 (4%) | 30 (15%) | 49 (24%) | 115 (57%) | 39 (19%) |
