# Supplementary material for: Perceived barriers related to testing, management and treatment of HCV infection among physicians prescribing opioid agonist therapy: The C‐SCOPE Study
Source: J Viral Hepat. 2019 Jun 11;26(9):1094–104. doi: 10.1111/jvh.13119 (PMC6771477; doi:10.1111/jvh.13119)
Supplement: Supplementary file 2 [file JVH-26-1094-s002.pptx]

## Slide 1
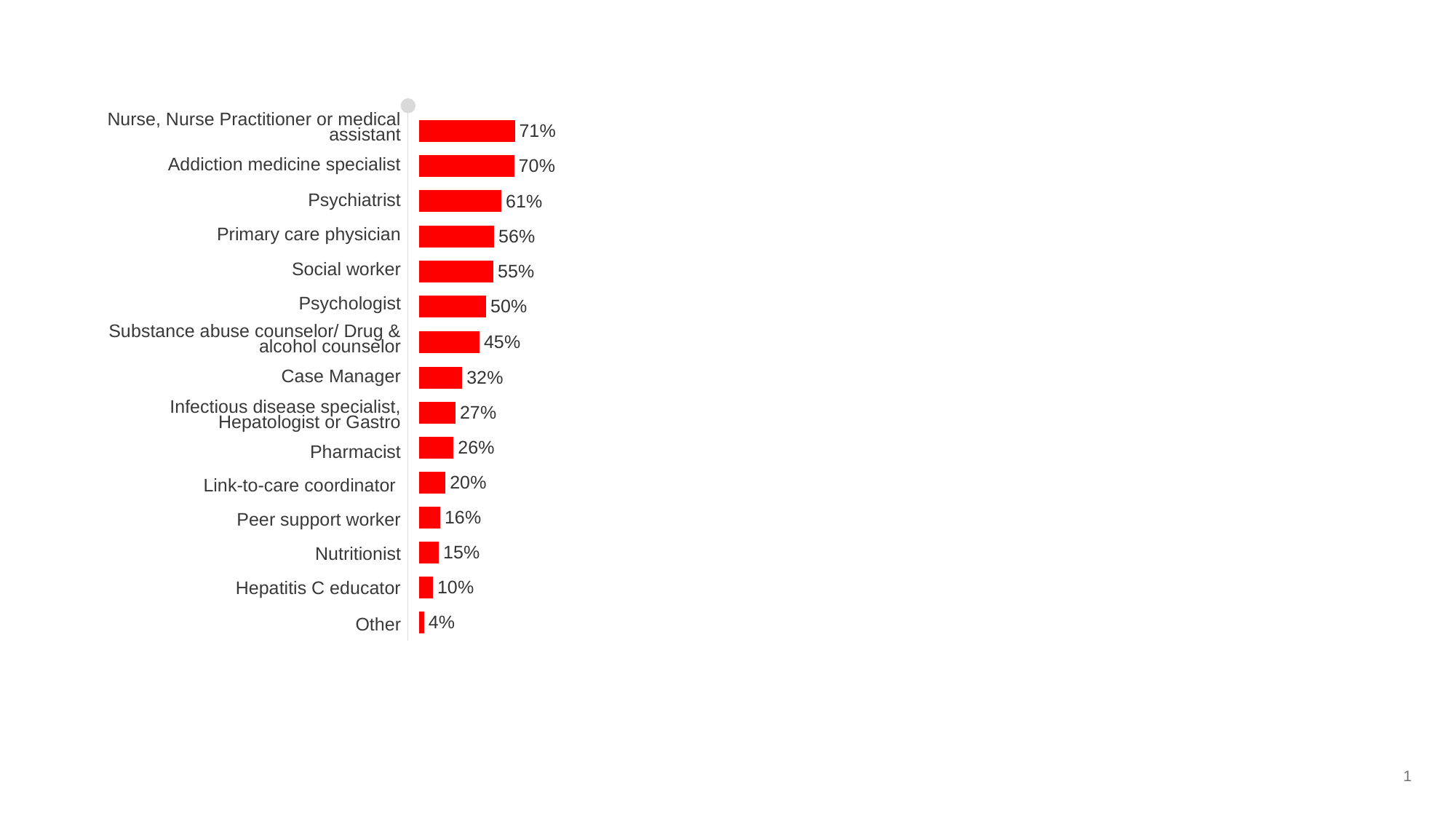

### Chart
| Category | total |
|---|---|
| Nurse, nurse practitioner or medical assistant | 0.71 |
| Addiction medicine specialist | 0.7044 |
| Psychiatrist | 0.6108 |
| Primary care physician | 0.5567 |
| Social worker | 0.5517 |
| Psychologist | 0.4975 |
| Substance abuse counselor/Drug and alcohol counselor | 0.4483 || Nurse, Nurse Practitioner or medical assistant |
| --- |
| Addiction medicine specialist |
| Psychiatrist |
| Primary care physician |
| Social worker |
| Psychologist |
| Substance abuse counselor/ Drug & alcohol counselor |
### Chart
| Category | total |
|---|---|
| Case manager (the individual whose primary responsibility is to manage the healthcare services given to a patient) | 0.32 |
| Infectious disease specialist, hepatologist or gastroenterologist | 0.2709 |
| Pharmacist | 0.2562 |
| Link-to-care coordinator (the individual whose primary responsibility is to work with patient to ensure he or she understand how and where to receive the appropriate medical care) | 0.197 |
| Peer support worker (an individual who has been treated or is currently receiving treatment for hepatitis C with the responsibilities that may include (1) encouraging new patients to engage in hepatitis C related services on- site through clinic outreach a | 0.1576 |
| Nutritionist | 0.1478 |
| Hepatitis C educator | 0.1034 |
| Other | 0.0394 || Case Manager |
| --- |
| Infectious disease specialist, Hepatologist or Gastro |
| Pharmacist |
| Link-to-care coordinator |
| Peer support worker |
| Nutritionist |
| Hepatitis C educator |
| Other |
