# Supplementary material for: Perceived barriers related to testing, management and treatment of HCV infection among physicians prescribing opioid agonist therapy: The C‐SCOPE Study
Source: J Viral Hepat. 2019 Jun 11;26(9):1094–104. doi: 10.1111/jvh.13119 (PMC6771477; doi:10.1111/jvh.13119)
Supplement: Supplementary file 3 [file JVH-26-1094-s003.pptx]

## Slide 1
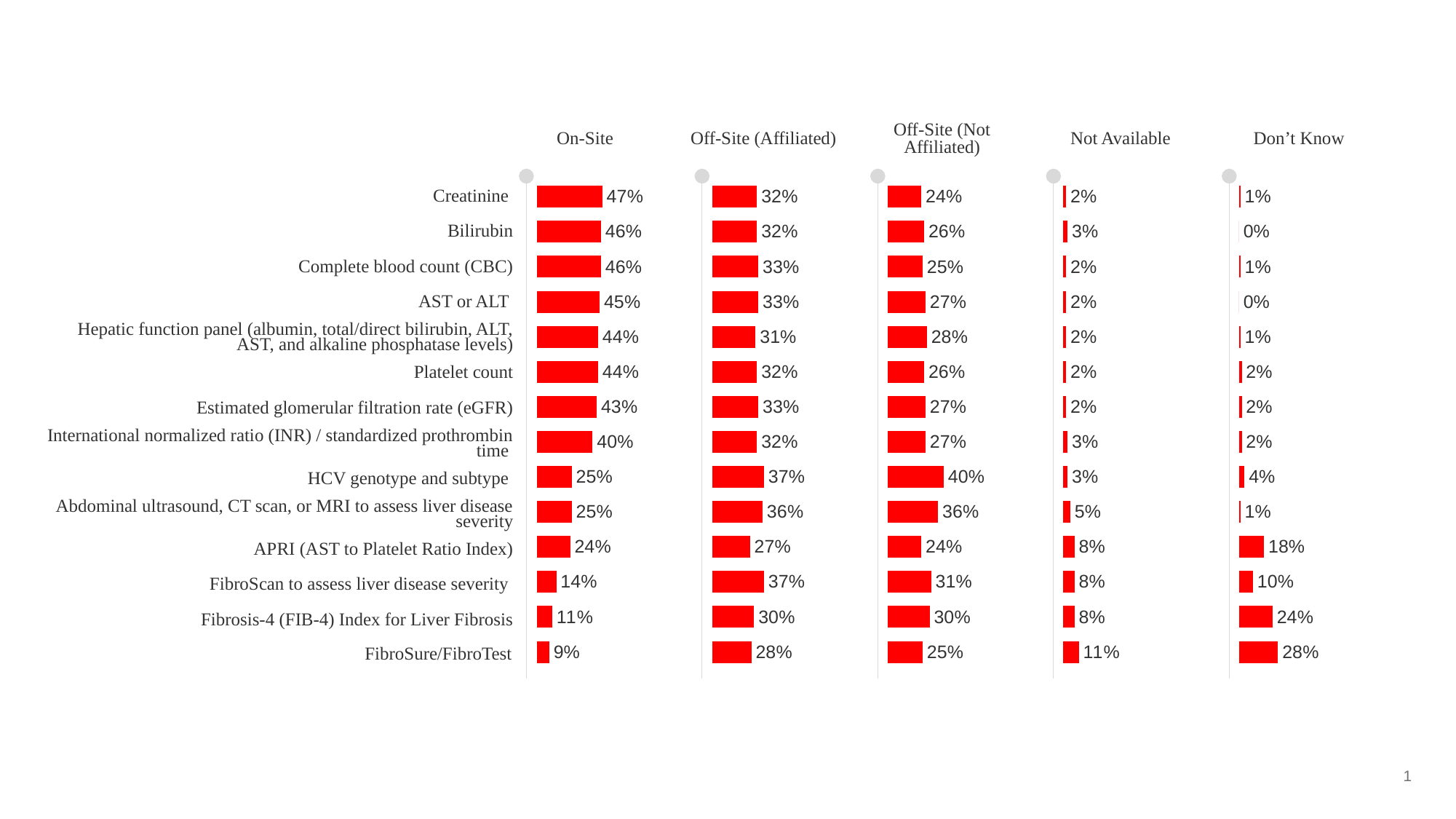

| On-Site | Off-Site (Affiliated) | Off-Site (Not Affiliated) | Not Available | Don’t Know |
| --- | --- | --- | --- | --- |
### Chart
| Category | On-Site |
|---|---|
| Creatinine | 0.47 |
| Bilirubin | 0.46 |
| Complete blood count (CBC) | 0.46 |
| AST or ALT | 0.45 |
| Hepatic function panel (albumin, total/direct bilirubin, ALT, AST, and alkaline phosphatase levels) | 0.44 |
| Platelet count | 0.44 |
| Estimated glomerular filtration rate (eGFR) | 0.43 |
| International normalized ratio (INR) / standardized prothrombin time | 0.4 |
| HCV genotype and subtype | 0.25 |
| Apbdominal ultrasound, CT scan, or MRI to assess liver disease severity | 0.25 |
| APRI (AST to Platelet Ratio Index) | 0.24 |
| FibroScan to assess liver disease severity | 0.14 |
| Fibrosis-4 (FIB-4) Index for Liver Fibrosis | 0.11 |
| FibroSure/FibroTest | 0.09 |
### Chart
| Category | Off-Site (Affiliated) |
|---|---|
| Creatinine | 0.32 |
| Bilirubin | 0.32 |
| Complete blood count (CBC) | 0.33 |
| AST or ALT | 0.33 |
| Hepatic function panel (albumin, total/direct bilirubin, ALT, AST, and alkaline phosphatase levels) | 0.31 |
| Platelet count | 0.32 |
| Estimated glomerular filtration rate (eGFR) | 0.33 |
| International normalized ratio (INR) / standardized prothrombin time | 0.32 |
| HCV genotype and subtype | 0.37 |
| Apbdominal ultrasound, CT scan, or MRI to assess liver disease severity | 0.36 |
| APRI (AST to Platelet Ratio Index) | 0.27 |
| FibroScan to assess liver disease severity | 0.37 |
| Fibrosis-4 (FIB-4) Index for Liver Fibrosis | 0.3 |
| FibroSure/FibroTest | 0.28 |
### Chart
| Category | Off-Site (Not Affiliated) |
|---|---|
| Creatinine | 0.24 |
| Bilirubin | 0.26 |
| Complete blood count (CBC) | 0.25 |
| AST or ALT | 0.27 |
| Hepatic function panel (albumin, total/direct bilirubin, ALT, AST, and alkaline phosphatase levels) | 0.28 |
| Platelet count | 0.26 |
| Estimated glomerular filtration rate (eGFR) | 0.27 |
| International normalized ratio (INR) / standardized prothrombin time | 0.27 |
| HCV genotype and subtype | 0.4 |
| Apbdominal ultrasound, CT scan, or MRI to assess liver disease severity | 0.36 |
| APRI (AST to Platelet Ratio Index) | 0.24 |
| FibroScan to assess liver disease severity | 0.31 |
| Fibrosis-4 (FIB-4) Index for Liver Fibrosis | 0.3 |
| FibroSure/FibroTest | 0.25 |
### Chart
| Category | Not Available |
|---|---|
| Creatinine | 0.02 |
| Bilirubin | 0.03 |
| Complete blood count (CBC) | 0.02 |
| AST or ALT | 0.02 |
| Hepatic function panel (albumin, total/direct bilirubin, ALT, AST, and alkaline phosphatase levels) | 0.02 |
| Platelet count | 0.02 |
| Estimated glomerular filtration rate (eGFR) | 0.02 |
| International normalized ratio (INR) / standardized prothrombin time | 0.03 |
| HCV genotype and subtype | 0.03 |
| Apbdominal ultrasound, CT scan, or MRI to assess liver disease severity | 0.05 |
| APRI (AST to Platelet Ratio Index) | 0.08 |
| FibroScan to assess liver disease severity | 0.08 |
| Fibrosis-4 (FIB-4) Index for Liver Fibrosis | 0.08 |
| FibroSure/FibroTest | 0.11 |
### Chart
| Category | Don’t Know |
|---|---|
| Creatinine | 0.01 |
| Bilirubin | 0.0 |
| Complete blood count (CBC) | 0.01 |
| AST or ALT | 0.0 |
| Hepatic function panel (albumin, total/direct bilirubin, ALT, AST, and alkaline phosphatase levels) | 0.01 |
| Platelet count | 0.02 |
| Estimated glomerular filtration rate (eGFR) | 0.02 |
| International normalized ratio (INR) / standardized prothrombin time | 0.02 |
| HCV genotype and subtype | 0.04 |
| Apbdominal ultrasound, CT scan, or MRI to assess liver disease severity | 0.01 |
| APRI (AST to Platelet Ratio Index) | 0.18 |
| FibroScan to assess liver disease severity | 0.1 |
| Fibrosis-4 (FIB-4) Index for Liver Fibrosis | 0.24 |
| FibroSure/FibroTest | 0.28 || Creatinine |
| --- |
| Bilirubin |
| Complete blood count (CBC) |
| AST or ALT |
| Hepatic function panel (albumin, total/direct bilirubin, ALT, AST, and alkaline phosphatase levels) |
| Platelet count |
| Estimated glomerular filtration rate (eGFR) |
| International normalized ratio (INR) / standardized prothrombin time |
| HCV genotype and subtype |
| Abdominal ultrasound, CT scan, or MRI to assess liver disease severity |
| APRI (AST to Platelet Ratio Index) |
| FibroScan to assess liver disease severity |
| Fibrosis-4 (FIB-4) Index for Liver Fibrosis |
| FibroSure/FibroTest |
